# Pain Relief after Stereotactic Radiotherapy of Pancreatic Adenocarcinoma: An Updated Systematic Review

**DOI:** 10.3390/curroncol29040214

**Published:** 2022-04-11

**Authors:** Milly Buwenge, Alessandra Arcelli, Francesco Cellini, Francesco Deodato, Gabriella Macchia, Savino Cilla, Erika Galietta, Lidia Strigari, Claudio Malizia, Silvia Cammelli, Alessio G. Morganti

**Affiliations:** 1 Radiation Oncology, IRCCS Azienda Ospedaliero-Universitaria di Bologna, 40138 Bologna, Italy; alessandra.arcelli2@unibo.it (A.A.); erika.galietta@studio.unibo.it (E.G.); silvia.cammelli2@unibo.it (S.C.); alessio.morganti2@unibo.it (A.G.M.); 2 Department of Experimental, Diagnostic and Specialty Medicine—DIMES, Alma Mater Studiorum University of Bologna, Via Albertoni 15, 40138 Bologna, Italy; 3 Istituto di Radiologia, Università Cattolica del Sacro Cuore, 00168 Roma, Italy; francesco.cellini@policlinicogemelli.it (F.C.); francesco.deodato@gemellimolise.it (F.D.); 4 Dipartimento di Scienze Radiologiche, Radioterapiche ed Ematologiche, Fondazione Policlinico Universitario A. Gemelli, IRCCS, UOC di Radioterapia, 00168 Roma, Italy; 5 Radiotherapy Unit, Gemelli Molise Hospital, Fondazione Policlinico Universitario A. Gemelli, IRCCS, Largo Agostino Gemelli 1, 86100 Campobasso, Italy; gabriella.macchia@unicatt.it; 6 Medical Physic Unit, Gemelli Molise Hospital, Fondazione Policlinico Universitario A. Gemelli, IRCCS, Largo Agostino Gemelli 1, 86100 Campobasso, Italy; savino.cilla@unicatt.it; 7 Medical Physics, IRCCS Azienda Ospedaliero-Universitaria di Bologna, 40138 Bologna, Italy; lidia.strigari@aosp.bo.it; 8 Nuclear Medicine, IRCCS Azienda Ospedaliero-Universitaria di Bologna, 40138 Bologna, Italy; claudio.malizia@aosp.bo.it

**Keywords:** radiotherapy, chemotherapy, pain, palliation, stereotactic radiotherapy, systematic review

## Abstract

Severe pain is frequent in patients with locally advanced pancreatic ductal adenocarcinoma (PDCA). Stereotactic body radiotherapy (SBRT) provides high local control rates in these patients. The aim of this review was to systematically analyze the available evidence on pain relief in patients with PDCA. We updated our previous systematic review through a search on PubMed of papers published from 1 January 2018 to 30 June 2021. Studies with full available text, published in English, and reporting pain relief after SBRT on PDCA were included in this analysis. Statistical analysis was carried out using the MEDCALC statistical software. All tests were two-sided. The I^2^ statistic was used to quantify statistical heterogeneity (high heterogeneity level: >50%). Nineteen papers were included in this updated literature review. None of them specifically aimed at assessing pain and/or quality of life. The rate of analgesics reduction or suspension ranged between 40.0 and 100.0% (median: 60.3%) in six studies. The pooled rate was 71.5% (95% CI, 61.6–80.0%), with high heterogeneity between studies (Q^2^ test: *p* < 0.0001; I^2^ = 83.8%). The rate of complete response of pain after SBRT ranged between 30.0 and 81.3% (median: 48.4%) in three studies. The pooled rate was 51.9% (95% CI, 39.3–64.3%), with high heterogeneity (Q^2^ test: *p* < 0.008; I^2^ = 79.1%). The rate of partial plus complete pain response ranged between 44.4 and 100% (median: 78.6%) in nine studies. The pooled rate was 78.3% (95% CI, 71.0–84.5%), with high heterogeneity (Q^2^ test: *p* < 0.0001; I^2^ = 79.4%). A linear regression with sensitivity analysis showed significantly improved overall pain response as the EQD2α/β:10 increases (*p*: 0.005). Eight papers did not report any side effect during and after SBRT. In three studies only transient acute effects were recorded. The results of the included studies showed high heterogeneity. However, SBRT of PDCA resulted reasonably effective in producing pain relief in these patients. Further studies are needed to assess the impact of SBRT in this setting based on Patient-Reported Outcomes.

## 1. Introduction

Pancreatic ductal adenocarcinoma (PDAC) is the fourth leading cause of cancer-related deaths and is projected to become the second leading cause of cancer mortality by 2030 [1]. Most patients present with metastatic or unresectable disease [2].

The prognosis is very poor in these settings, and the quality of life is significantly worsened by symptoms such as pain, jaundice, fatigue, weight loss/cachexia, and ascites [3]. In particular, abdominal and/or mid-back pain is reported by 70–80% of patients with advanced PDCA [3,4] and is difficult to treat suggesting a multifactorial pathophysiology. Notably, PDCA-related pain management is often suboptimal with patients often being undertreated [5].

PDCA pain management is generally based on drug therapy (opioids, gabapentine, duloxetine) and, depending on the pain pathogenesis, also several local procedures (celiac plexus block, neurolysis, high-intensity focused ultrasound, endoscopic stents) can be used [3,6,7]. However, both drug treatment and celiac plexus neurolysis have limited efficacy and are associated with side effects and possible complications [5,8,9]. Radiation therapy is another therapeutic option in PDCA palliation. The available studies are few but consistently reported over 50% pain relief rates [10,11,12].

In the last 15 years, in addition to standard radiotherapy or chemoradiation of PDCA, stereotactic body radiotherapy (SBRT) became available. Advantages of the latter are high conformation of the dose and thus potential reduction of toxicity, high biological efficacy due to the concentration over time, and short treatment duration which facilitates integration with systemic treatments [13].

Three years ago, we published a systematic review on pain relief after SBRT in PDCA [14]. The literature search included papers published from January 2000 to December 2017. The results showed a large variability that prevented an evaluation of factors correlated with a better palliative effect (dose, fractionation, therapeutic combinations). However, from January 2018 to December 2020, 180 papers on SBRT of PDCA were recorded in PubMed [15].

Given this large availability of new evidence, we decided to update our previous systematic review. The aim of the updated analysis was to provide more homogeneous results and clearer indications on the most effective SBRT modalities to achieve pain relief in this setting. Therefore, the purpose of this analysis is to report the results of our updated literature review on SBRT efficacy in PDCA pain relief.

## 2. Results

### 2.1. Search Results

**Figure 1:**
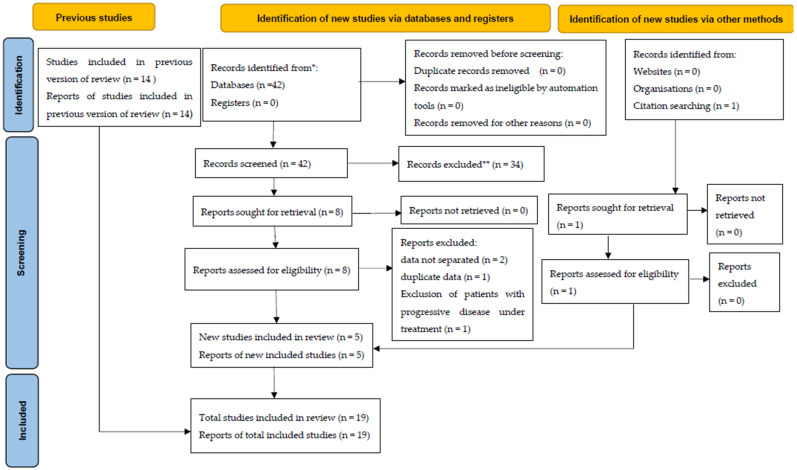
PRISMA 2020 flow diagram for updated systematic reviews. (*: database: Medical Subject Headings PubMed platform; **: automation tools not used for paper exclusion).

In our updated literature analysis, we included 19 papers [16,17,18,19,20,21,22,23,24,25,26,27,28,29,30,31,32,33,34]. In fact, six new papers were identified and added to the 14 reports included in the previous analysis. However, given that one study [35] reported the same data as a paper already included in the analysis [25], it was excluded from the list of evaluable studies. (Figure 1) Overall, the analyzed papers included 615 patients, of whom 296 reported pain before SBRT. The characteristics of the analyzed studies in terms of pain relief are summarized in Table 1. Detailed data on study design, patients, tumors, treatment, pain relief, and toxicity are shown in the Supplementary Materials (Table S1).

### 2.2. Pain Relief

**Figure 2:**
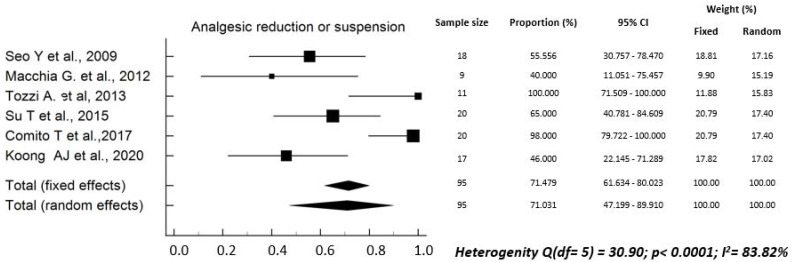
Overall rate of reduction or suspension of analgesic therapy [17,22,24,26,28,34].

**Figure 3:**
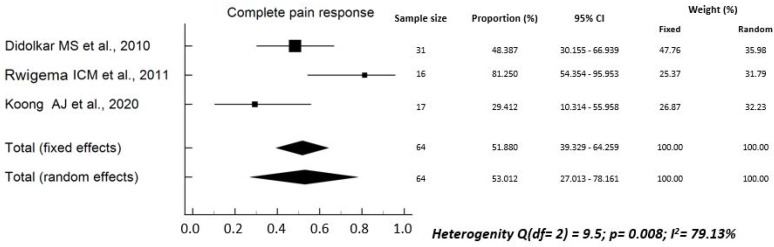
Overall complete response to pain [18,21,34].

**Figure 4:**
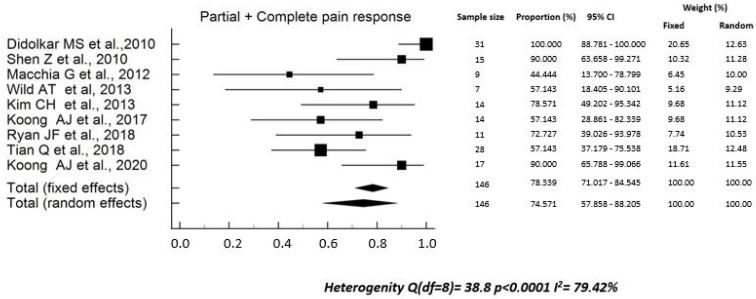
Overall global (complete *plus* partial) response to pain [18,19,22,23,27,29,31,32,34].

The rate of analgesics reduction *or* suspension ranged between 40.0% and 100.0% (median: 60.3%) in six studies [17,22,24,26,28,34]. The pooled rate was 71.5% (95% CI, 61.6–80.0%), with high heterogeneity between studies (Q^2^ test: *p* < 0.0001; I^2^ = 83.8%) (Figure 2). The rate of complete pain response after SBRT ranged between 30.0% and 81.3% (median: 48.4%) in three studies [18,21,34]. The pooled rate was 51.9% (95% CI, 39.3–64.3%), with high heterogeneity (Q^2^ test: *p* < 0.008; I^2^ = 79.1%) (Figure 3). The rate of partial *plus* complete pain response ranged between 44.4% and 100% (median: 78.6%) in nine studies [18,19,22,23,27,29,31,32,34]. The pooled rate was 78.3% (95% CI, 71.0–84.5%), with high heterogeneity (Q^2^ test: *p* < 0.0001; I^2^ = 79.4%) (Figure 4).

Three studies in which more than 90% of patients were treated with SBRT plus chemotherapy reported 90% median pain relief rate (range. 44.4–100%) [19,23,36] while in four studies in which less than 30% of patients received chemotherapy in combination with SBRT, the median pain relief was 67.8% (range: 57–90%) [20,24,28,33].

No studies reported the analgesic results of SBRT differentiating them according to the tumor site in the pancreas (head vs. body vs. tail).

Some studies where a low dose of radiation was delivered (median EQD2α/β:10: 31.3–50.0 Gy, median: 31.3 Gy) showed relatively poor results in terms of pain relief (CR plus PR rate: 44.4–57.1%) [22,23,29,32]. In contrast, authors using intermediate doses (median EQD2α/β:10: 68.0–79.7 Gy, median: 70.4 Gy) reported better results in terms of pain palliation (CR plus PR rates: 78.6–90.0%) [19,21,27,34].

### 2.3. Impact of Radiotherapy Dose on Pain Relief

**Figure 5:**
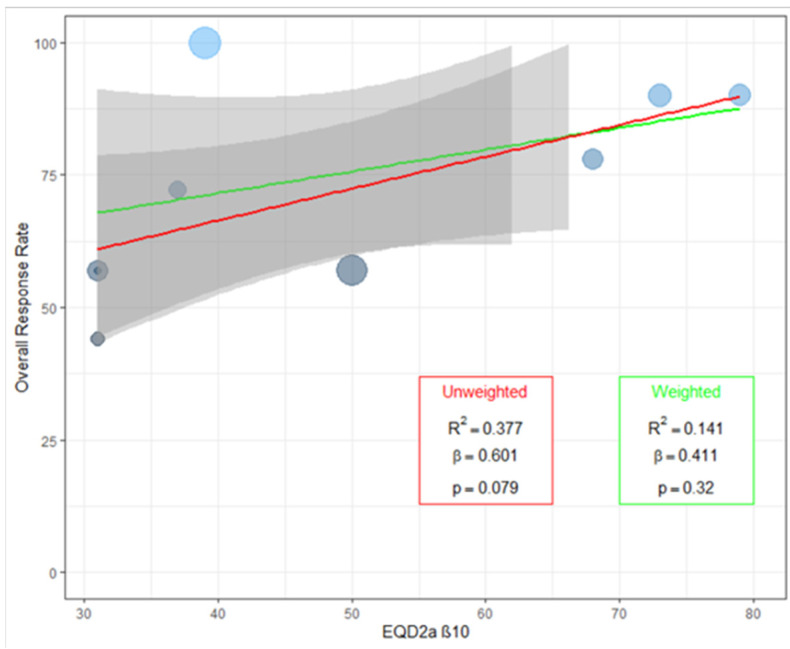
Linear regression: equivalent doses in 2-Gy fractions (EQD2) versus overall response rate of pain.

The linear regression of overall response rate of pain after SBRT, based on the equivalent dose in 2 Gy per fraction (EQD2, α/β ratio *=* 10), showed a positive trend with increased doses, but only when the analysis was performed without weighting the results based on the number of patients in the individual case series (Figure 5). Furthermore, it was clear the presence of an outlier study (18), the only paper reporting 100% overall response rate. Therefore, the analysis was repeated, after removing this result, and showed a highly significant positive effect of higher EQD2 on pain relief (*p*: 0.004 and *p*: 0.005 for unweighted and weighted analysis, respectively) (Figure S1).

### 2.4. Pain-Free Survival

Sixteen studies did not report on the pain control duration [17,19,20,21,22,23,24,25,26,27,28,29,31,32,33,34]. One study, which showed a worsening of pain 14 days after SBRT, showed that 50% of patients had a reduction of this symptom three months after SBRT [16]. In another study, it was observed that patients with complete pain relief remained symptom free after six months [18]. Finally, another study confirmed that SBRT-induced pain reduction was stable after at least six months [30].

### 2.5. Toxicity

Seven papers did not report any side effect during and after SBRT [19,20,24,26,27,28,33]. In three studies only transient acute effects were recorded [21,29,30]. The incidence of acute/late effects was 7.4% in one study [34]. Other studies reported variable incidences of late side effects (gastrointestinal bleeding, obstruction, bleeding, perforation) (Table S1) [16,17,18,22,23,25,31,32].

The only study where cases of gastric perforation were recorded was the one where the highest SBRT doses were delivered in terms of EQD2α/β:10 and EQD2α/β:3 (93.8 Gy and 162.0 Gy, respectively) [16]. In studies where neither acute nor late effects were recorded, EQD2α/β:10 ranged between 50.0 and 79.7 Gy (median: 65.5 Gy) and EQD2α/β:3 ranged between 78.0 and 135.0 Gy (median: 95.0 Gy) [19,20,24,26,27,28,33].

### 2.6. Quality Assessment of the Analyzed Studies

**Figure 6:**
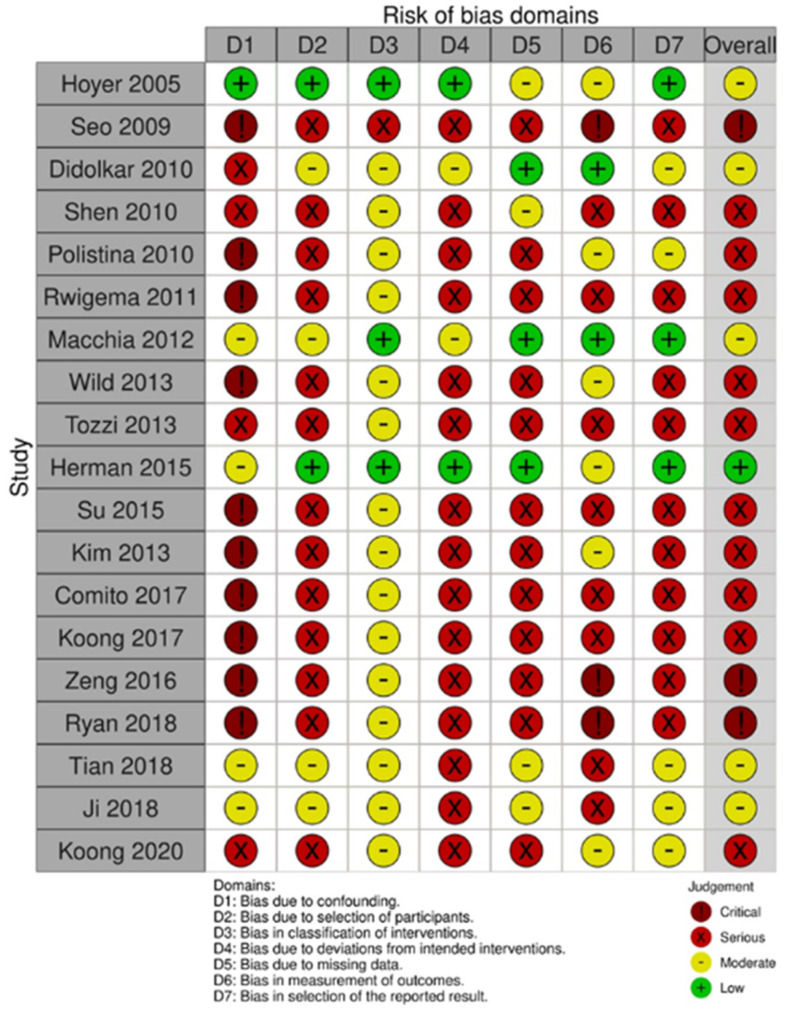
Risk of Bias in Non-Randomized Studies—of Interventions (ROBINS-I) traffic-light plot [16,17,18,19,20,21,22,23,24,25,26,27,28,29,30,31,32,33,34].

**Figure 7:**
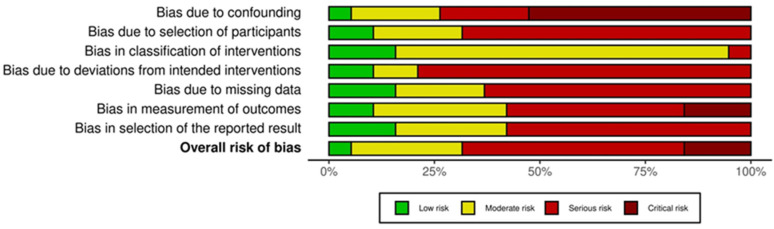
Risk of Bias in Non-Randomized Studies—of Interventions (ROBINS-I) summary plot.

Most studies included in this review presented a critical (3 reports) [17,30,31] or serious (10 reports) risk of bias [19,20,21,23,24,26,27,28,29,34]. The domains with the highest critical-serious risk of bias were “bias due to confounding” and “bias due to deviations from intended intervention”. The risk of critical-serious bias was greater than 50% for all domains. The Traffic-Light plot and the Summary plot based on the risk of bias in non-randomized studies of intervention (ROBINS-I) tool are shown in Figure 6 and Figure 7, respectively. The quality of evidence, considering pain relief as the outcome and based on the GRADE assessment, was high, moderate, low, and very low in one, three, eight, and seven papers, respectively (Table 1).

## 3. Discussion

Pain is a frequent symptom in PDCA patients with a very negative impact on patients’ quality of life. Many treatments can reduce this symptom, but in most cases, there are barriers that limit their use. For example, the use of chemotherapy is hindered by the poor performance status, at least in some categories of patients, similarly to what happens for radiotherapy or chemoradiation with conventional fractionation. Furthermore, the use of opioid drugs is limited by side effects and concern for abuse. Finally, interventional gastroenterologists, although available at academic centers, may not be available in the general community for neurolysis, high-intensity focused ultrasound, and endoscopic pancreatic stenting procedures.

In this regard, SBRT represents a potentially useful treatment in patients with PDCA-induced pain. Indeed, some studies showed improved local control in PDCA after SBRT compared to standard chemoradiation [36,37]. Furthermore, in this setting of patients with limited survival expectancy, the reduction of acute side effects demonstrated in some comparisons with chemoradiation represents an important advantage in terms of quality of life [37,38].

Despite this background, there are no studies in the literature specifically aimed at the analgesic effect of SBRT in PDCA. Furthermore, to the best of our knowledge, our analysis remains the only systematic review on this topic. This updated review was not based on automatic monitoring systems of available trials, but on the simple observation of the growing number of studies published on SBRT of PDCA. In fact, we decided to repeat this systematic review having noticed that the number of reports on the PDCA’s SBRT had increased by over 180 papers since the publication of our previous analysis [14]. However, by including the term “pain” in our research, the number of reports was reduced to just 42 papers. Even considering the limitations of this study, mainly resulting from the low level of available evidence, our analysis suggests that SBRT is effective in achieving pain relief in most PDCA patients. Furthermore, some studies suggest that pain relief is long-lasting in these patients [16,18,30].

In fact, only two studies, enrolling a total of 45 patients, reported a lack of improvement in pain [20] or even its worsening [16]. In this regard, some observations can be made. In the first study a comparison between pain (measured with a visual analog scale) before and after SBRT was performed on all treated patients including those without pain before therapy. Obviously, this type of analysis may prevent the detection of improvement in patients with pain [20]. Regarding the second paper, it should be emphasized that a very large planning target volume was defined by including the peritumoral edema plus a margin of up to 10 mm. Furthermore, in this study, the Equivalent Dose in 2 Gy per fraction, calculated using an α/β ratio of 10 (EQD2_α/β:10_), was the highest (93.8 Gy) among the studies included in our analysis. Therefore, it is possible that the combination of very high dose and very large, irradiated volume may have caused complications in the gastrointestinal tract producing local pain. In fact, the toxicity recorded in that analysis was the most serious of all the reports, with duodenal mucositis/ulcerations (18%) and gastric perforations (4.5%) [16]. Therefore, the latest study suggests a detrimental effect of very high doses on the palliative impact of SBRT in this setting. On the contrary, our analysis on the impact of EQD2 on the overall pain response showed, in particular in the sensitivity analysis, a significantly improved response rate with the dose increase. The removal of an outlier study from the analysis may be questionable. However, it should be noted that the authors of that study reported partial or complete pain relief in 31 patients undergoing SBRT but did not specify the timing of the assessment. Furthermore, looking at the survival curve after SBRT in their paper, the immediate descending part of the graph is clear, making it highly unlikely that all treated patients could have been assessed for symptomatic response.

Even concurrent chemoradiation with standard fractionation can improve cancer-related pain [39]. However, an assessment of the treatment impact on pain was not performed in direct comparisons between chemoradiation and SBRT [36,37,38,40,41,42,43]. Moreover, chemotherapy alone is able to reduce PDCA pain [44]. However, even direct comparisons between SBRT and chemotherapy or between chemotherapy alone and chemotherapy combined with SBRT in terms of pain relief are lacking. Nonetheless, it should be noted that, in the two studies reporting pain control over six months after SBRT, all patients underwent adjuvant chemotherapy [18,30]. However, comparing the median pain relief after SBRT, in case series in which chemotherapy was administered in the minority [20,24,28,33] or in the majority [19,23,36] of patients, no clear differences were found regarding this endpoint (67.8% and 90%, respectively). Anyway, it is difficult to compare the results of SBRT in pain relief with those obtained with other systemic or local therapies. For example, it is likely that, at least in some centers, patients without lymph node metastases and without extension to hollow organs of the gastrointestinal tract, such as stomach and duodenum, have been preferentially referred to SBRT rather than chemoradiation and/or chemotherapy. Clearly, these patients, with more localized cancer, would be less likely to have severe pain and more likely to obtain a palliative benefit

Similarly, no direct comparisons are available between SBRT and local analgesic treatments. Only one paper, included in our analysis, compared SBRT versus SBRT combined with celiac block [33]. In fact, Ji et al. showed that the average and worst pain, measured using a Numerical Rating Scale, were significantly lower after SBRT compared to baseline values (*p* < 0.05). However, there was a significant decrease (*p* < 0.05) in average Numerical Rating Scale in the SBRT plus celiac plexus block group compared to SBRT alone group at two, three and four weeks after SBRT. Moreover, the worst Numerical Rating Scale in the SBRT plus celiac plexus block group was significantly lower (*p* < 0.05) compared to the SBRT alone group at three and four weeks after SBRT [33]. The results of this study seem to suggest that the analgesic effect of SBRT can be improved through the combination with other local treatments.

Our study has several limitations: (i) the sample size was small, with only three studies including more than 50 patients; (ii) in addition, some authors reported only the “p” value of the statistical significance of pain changes, without reporting the percentages of subjects with pain before and after SBRT [16,20,25,30,33]. Therefore, we had to exclude these studies from the quantitative analysis to calculate the pooled values, as evident from Figure 2, Figure 3 and Figure 4. Particularly, for the same reasons, the only study reporting negative results in terms of pain relief could not be included. In fact, neither the rates of analgesic reduction or suspension, nor the rates of partial plus complete pain response after SBRT were reported in the paper by Hoyer et al. [16]; (iii) in most cases, the studies were retrospective or case series and therefore with high risk of under-reporting the incidence of side effects; (iv) due to the same reasons, most studies included in this review presented a critical (3 reports) [17,30,31] or serious (10 reports) [19,20,21,23,24,26,27,28,29,34] risk of bias; (v) the inclusion criteria were different between the different studies and the treatment modalities were very inhomogeneous in terms of planning and delivery techniques, definition of the planning target volume, dose and fractionation, and integration with chemotherapy; (vi) the criteria for evaluating pain relief were different between different series, and the duration of response of this symptom was evaluated only in a minority of analyses.

## 4. Materials and Methods

This analysis was conducted based on the Preferred Reporting Items for Systematic reviews and Meta-Analyses (PRISMA) statement [45]. The research question was framed using the Population, Intervention, Comparison and Outcomes (PICO) method as follows: “In patients with unresected pancreatic adenocarcinoma (P), is stereotactic radiotherapy (I), compared with standard treatments—chemotherapy, chemoradiation—(C), effective in terms of pain relief (O)?”

### 4.1. Endpoints

The primary endpoint of this updated analysis was pain relief. It was analyzed in terms of symptom’s response (partial or complete) and based on reduction or suspension of analgesics intake. Other evaluated endpoints were pain-free survival and acute and late toxicity.

### 4.2. Selection Criteria

Studies with full available text, published in English, and reporting pain relief after SBRT of PDCA were included in this analysis. Moreover, only patients undergoing SBRT on primary PDAC were included in the analysis. Studies were excluded in the case of abstracts of conference proceedings; case reports; inclusion of non-adenocarcinoma pancreatic cancers PDCA, SBRT delivered in >10 fractions, SBRT delivered over an entire lymph node region, and PDCA irradiated with a technique different from SBRT; studies on animal models/preclinical studies; planning studies; imaging studies; study protocols; pain relief not separately reported for PDCA; systematic or narrative reviews; meta-analyses; letter-commentaries-editorials; surveys; exclusion from the analysis of patients not completing SBRT or with progressive disease during treatment; guidelines–recommendations; or duplicate data. In case of inclusion of the same patients in subsequent publications, the most complete or recent article was selected.

### 4.3. Literature Search and Data Extraction

We updated on the Medical Subject Headings PubMed platform the literature search performed in our previous paper [14] on 30 January 2021. Additionally, the research was further updated on 30 June 2021. The same search strategy was adopted: (“pancreatic neoplasms” [MeSH Terms]) OR (“pancreatic” [All Fields] AND “neoplasms” [All Fields]) OR “pancreatic neoplasms” [All Fields] OR (“pancreatic” [All Fields] AND “cancer” [All Fields]) OR (“pancreatic cancer” [All Fields]) AND (“radiotherapy” [Subheading] OR “radiotherapy” [All Fields] OR “radiotherapy” [MeSH Terms]) AND (“pain” [MeSH Terms] OR “pain” [All Fields]). Only papers published from 1 January 2018 to 31 December 2020 were included. Two authors (MB, AA) selected papers from titles and abstracts. Subsequently, the same authors independently evaluated the selected papers at text level to verify their suitability for the analysis. Finally, selected papers were evaluated to extract the data useful for the review. Two authors (MB, AA) independently extracted the data, and any discrepancies were solved discussing with the senior authors (SiC, AGM).

### 4.4. Statistical Analysis

The statistical analysis was carried out using the MEDCALC statistical software (version 15.2.2, MedCalc Software bvba, Ostend, Belgium). All tests were two-sided. The I^2^ statistic was used to quantify statistical heterogeneity (high heterogeneity level: >50%). The latter was tried out with the Q^2^ test (significance level: *p* < 0.1), and statistical significance was considered as *p* < 0.05, except when investigating heterogeneity among studies (*p* < 0.1). In case of heterogeneity among selected studies, rates and proportions were pooled using a random-effects model. A fixed-effect model was used in other cases. The dependent variables were modelled on the logit (log-odds) scale, converted back to percentages, and then presented as point estimates and 95% CI. The impact of the equivalent dose in fractions of 2 Gy (EQD2, α/β = 10) was performed by linear regression, with and without weighting based on the sample size of the individual case series. In addition, a sensitivity analysis was planned with the exclusion of any outliers.

### 4.5. Quality Assessment

Since no randomized trial was conducted on the topic of this analysis, the assessment of the risk of bias was performed using the risk of bias in non-randomized studies of intervention (ROBINS-I) tool [46]. It includes risk of bias due to confounding factors, selection of participants into the study, classification of interventions, deviations from intended intervention, missing data, measurement of outcomes, and selection of the reported results. Two authors (MB, AA) ranked independently the included papers and resolved any disagreement by discussion. The results of this analysis were reported graphically using the robvis tool [47]. The quality of evidence, considering pain relief as the outcome, was based on the GRADE assessment [48].

## 5. Conclusions

SBRT is reasonably effective in pain relief of PDCA patients. Very high doses of SBRT can have a detrimental effect while intermediate doses seem more effective than low doses. Particular attention to treatment details is needed considering the risk of side effects, sometimes of not negligible severity. Finally, since surgical resection is the only possibility of cure for patients with PDCA, the objectives of SBRT in patients with inoperable PDCA should be both survival prolongation and quality of life improvement.

Therefore, future studies should be aimed at: (i) prospective analyses including a systematic assessment of the impact of SBRT on pain and more generally on quality of life; (ii) comparison between the impact on pain of SBRT and chemoradiation; (iii) comparison between pain control achievable with systemic therapies alone or with systemic therapies combined with SBRT; (iv) identification of the best combination between SBRT and systemic therapies not only in terms of outcome but also of palliation of symptoms including pain; (v) identification of the best combinations between SBRT and local pain treatments (celiac plexus block, neurolysis, high-intensity focused ultrasound) to achieve the highest and most lasting relief from this symptom.

## Figures and Tables

**Table 1:** Characteristics of analyzed studies and outcomes.

Author, Year	Patients (with Pain)	Pain Evaluation Methods	Pain Response Outcomes	Quality of Evidence (GRADE)
Hoyer et al., 2005 [16]	22 (15)	Scale: WHO; Timing: baseline and after treatment (14 days, 2 and 3 months)	First evaluation after treatment: significant worsening of pain (*p* = 0.008) and increased (non-significant) use of analgesics (*p* = 0.08). Evaluation at 3 months: pain reduction in 50% of patients.	Low
Seo et al., 2009 [17]	30 (18)	Pain relief was evaluated by comparing analgesic consumptions before and after SBRT.	Analgesic consumption was reduced in 10 patients (55.6%) after SBRT.	Low
Didolkar et al., 2010 [18]	85 (moderate-severe: 31)	Scale: 0–10	Analysis performed in patients with pain score > 4: Complete pain relief (for at least 6 months): 48.4%, Partial pain relief: 51.6%.	Very low
Shen et al., 2010 [19]	20 (15)	Scale: visual analog with numeric rating	Some degree of “pain relief” was recorded in 90% of treated patients	Low
Polistina et al., 2010 [20]	23 (NR)	Scale: visual analog with numeric rating; recording of analgesic use.	Mean pretreatment pain score: 3.91 ± 2.41; mean post-treatment score (three months): 3.65 ± 2.81 [*p* > 0.05]). The authors reported a reduction in the consumption of analgesic drugs, but without showing the data.	Very low
Rwigema et al., 2011 [21]	71 (16)	NR	Complete pain relief: 81.3%	Very low
Macchia et al., 2012 [22]	16 (9)	Scales: visual analog with numerical rating; Pain score (symptom severity × frequency); Drug score (class of analgesics × frequency of intake)	Complete or partial pain relief: 44.4% Pain worsening: 6.2% Reduction in analgesic consumption: 40.0%	Moderate
Wild et al., 2013 [23]	18 (7)	NR	Pain relief: 57.1% (4–8 weeks after SBRT)	Very low
Tozzi et al., 2013 [24]	30 (11)	Scale: numerical rating score	Discontinuation of analgesic consumption: 63.6% Reduction in analgesic consumption: 36.4%	Very low
Herman et al., 2015 [25]	49 (NR)	Scale: QLQ-PAN26	Pain reduction from score 25 to score 17 (median values), statistically significant (*p* = 0.001), recorded 4 weeks after treatment.	High
Su et al., 2015 [26]	25 (20)	Scale: numerical rating score	Discontinuation of analgesic consumption: 50.0% Reduction in analgesic consumption: 15.0%	Low
Kim et al., 2013 [27]	26 (14)	NR	Abdominal pain relief: 80.0% Back pain relief: 75.0% Discontinuation of opioid consumption: 35.7%	
Comito et al., 2017 [28]	31 (22)	Scale: numerical rating score	Discontinuation of analgesic consumption: 59.1% Reduction in analgesic consumption: 40.9%	Low
Koong et al., 2017 [29]	23 (14)	NR	Complete pain relief: 57.1% Pain worsening: 7.1%	Low
Zeng et al., 2016 [30]	24 (13)	Scale: visual analog with numeric rating	Both short-term (1 week after SBRT: 2.7 ± 1.3) and long-term (6 months after SBRT: 1.2 ± 1.4) mean pain scores were significantly reduced compared to pre-treatment values (7.2 ± 2.5) (*p* < 0.05)	Low
Ryan et al., 2018 [31]	29 (11)	Scale: National Cancer Institute Common Terminology Criteria for Adverse Events	Pain relief: 72.7% (3 months after SBRT)	Very low
Tian et al., 2018 [32]	31 (28)	Scale: Brief Pain Inventory questionnaires (before treatment and one and three months after treatment)	Pain relief: 57.0% (1 month after SBRT)	Low
Ji et al., 2018 [33]	35 (35)	Scale: numerical rating score	Both mean and worst pain scores were significantly improved after SBRT (*p* < 0.05), in all assessments over time. Pain relief was further improved in a subset of patients undergoing both SBRT and celiac plexus block in the 2–4 weeks post-treatment interval.	Moderate
Koong et al., 2020 [34]	27 (17)	Scale: Stanford Pain Score	Complete pain relief: 30.0% Partial pain relief: 70.0%	Moderate

Abbreviations: NR: not reported; SBRT: stereotactic body radiation therapy.

## Supplementary Materials

The following supporting information can be downloaded at: https://www.mdpi.com/article/10.3390/curroncol29040214/s1. Table S1: detailed characteristics of study design, patients, tumors, treatment, pain relief, and toxicity. Figure S1: Linear regression with sensitivity analysis (removal of an outlier: Didolkar et al., 2010) of equivalent total doses in 2-Gy fractions versus overall response rate of pain.
