# Supplementary material for: Pain Relief after Stereotactic Radiotherapy of Pancreatic Adenocarcinoma: An Updated Systematic Review
Source: Curr Oncol. 2022 Apr 11;29(4):2616–29. doi: 10.3390/curroncol29040214 (PMC9032429; doi:10.3390/curroncol29040214)
Supplement: Supplementary file 1 [file curroncol-29-00214-s001.zip › curroncol-1575127-supplementary.pdf]

Current Oncology

MDPI

SUPPLEMENTARY

Pain relief after stereotactic radiotherapy of pancreatic cancer: an updated systematic review.

Table S1. detailed characteristics of study design, patients, tumors, treatment, pain relief, and toxicity. .

author, year	study design	inclusion criteria	pts [with pain]	stage	technique	PTV	dose prescription	RT dose, Gy median (range)	Median EQD2 α/β: 10	Median EQD2 α/β: 3	% of pts receiving chemo*	pain response rate, % [criteria]	pain-free survival (months)	grade 3-4 toxicity % (scale)
Hoyer M et al., 2005 [16]	phase I-II	LA	22 [15]	$T_{1-3}N_0M_0$	SBRT with standard LINAC	GTV + edema + 5-10 mm	PTV encompassed by 67% isodose	45/3 Fr.	93.8	162.0	0.0	increased pain after 14 days (p:0.008) [WHO toxicity scale]	reduced pain after 3 months in 50% of pts	gastric-duodenal mucositis/ulceration: 18.0 gastric perforation: 4.5 (WHO)
Seo Y et al., 2009 [17]	phase I	LA, no duodenal invasion, < 3 N+	30 [18]	T_4: 100.0% N_1: 30.0%	RRS	GTV + 2 mm, (4 mm CC)	to isodose covering 97% of PTV	40 in 2 Gy/Fr. (3D-CRT) + 16.5 (14-17); SF RS boost	76.4	104.4	70.0* (6 before RT; 15 concurrent to 3D-CRT)	RAA: 55.6 [comparison between analgesics consumption before versus after SBRT]	NR	acute: duodenal obstruction (3.3); late: 0.0 (RTOG)
Didolkar MS et al.,2010 [18]	retrosp	LA	85 [31 pts with pain score > 4/10]	LA M_1: 24.7†	RSBRT	GTV + 3 mm	to 80% isodose	25.5/3 Fr. (15-30)	39.3	58.6	100.0 after	in pts with pain score > 4: CR: 48.4 PR: 51.6‡ [0-10 scale]	CR lasted > 6 months	acute: duodenitis (14.1) gastritis (12.9) diarrhea (3.5); late: hemorrhage /obstruction (8.2) (CTC 2.0)

Shen Z et al., 2010 [19]	case series	LA	20 [15]	stage II-III	RSBRT	GTV + 3-5 mm	$V_{75\%}$ > 95%	45(32-55)/4 (3-6) Fr.	79.7	128.2	0.0	"some degree of pain relief": 90.0 [VAS]	NR	acute: 0.0 late: 0.0 (RTOG)
Polistina F et al., 2010 [20]	case series	LA < 6 cm	23 [NR]	N_1: 60.8%	RSBRT	NR	NR	30/3 Fr.	50.0	78.0	100.0* and after	no significant reduction of pain [VAS]	NR	acute: 0.0 late: 0.0 (CTC 3.0)
Rwigema ICM et al., 011 [21]	retrosp	LA or M1 §	71 [16]	LA:56 Rec: 16 M_1: 11 R_1: 17	RRS or LINAC	GTV + 2 mm	to 80-89% isodose	24 SF (18-25)	68.0	129.6	90.0* and/or after	CR: 81.3 [NR]	NR	acute GI: 4.2 late: 0.0 (NR)
Macchia G et al., 2012 [22]	phase I	LA	16 [9]	T_4: 87.5%‖ Rec: 12.5%‖	SBRT with standard LINAC	GTV + ≥ 10 mm	ICRU 62	25/5 Fr. (20-35)	31.3	40.0	100.0*	CR + PR: 44.4 Increased pain: 11.1 RAA: 40.0 [pain score, drug score, VAS]	NR	acute: 0.0 late: duodenal bleeding: 6.2 (RTOG)
Wild AT et al., 2013 [23]	retrosp	LA or Rec (previous RT)	18 [7]	LA: 16.7% Rec: 83.3%	SBRT	ITV + 1-3 mm	to isodose surrounding PTV	25/5 Fr. (20-27)	31.3	40.0	28.0 after	"Pain relief": 57.0 [NR]	NR	acute: 0.0 late: small bowel obstruction: 6.0 (NR)
Tozzi A et al., 2013 [24]	case series	LA or Rec ≤ 5 cm	30 [11]	LA: 70% Rec: 0%	VMAT FFF	GTV + 5 mm (10 mm CC)	CTV $V_{95\%}$ = 100%	45/6 Fr. (36-45)	65.5	95.0	100.0*	SAA: 63.6 RAA 36.4 [NRS]	NR	acute: 0.0 late: 0.0 (CTC 3.0)
Herman JM et al., 2015 [25]	phase II	LA	49 [NR]	NR	SBRT (VMAT)	GTV + 2-3 mm	$V_{100\%}$ > 90%	33/5 Fr.	45.7	63.4	90.0* 100.0 after	reduction of 8 points from baseline (25 points) [QLQ-PAN 26]	NR	acute: duodenal ulcer (2.0¶) late: duodenal ulcer/ bleeding: 6.4 (NR)
Su T et al., 2015 [26]	retrosp	LA or M1	25 [20]	LA: 25 M_1: 16	RSBRT	GTV + 1-2 mm	$V_{93\%}$ > 97	36/3 Fr. (30-48	66.0	108.0	8.0[f]* 8.0 after	SAA: 50.0 RAA: 15.0 [NRS]	NR	acute: 0.0 late: 0.0 (NR)

								in 3-4 Fr.)						
Kim CH et al., 2013 [27]	retrosp	not surgical candidates	26 [14]	stage I-IV	LINAC or RRS or RSBRT	GTV + 2 mm	to 80 – 93% isodose	24 (24-36)/1-3 Fr.	68.0	135.0	15.0* 23.0 after	pain relief: 78.6 SAA: 35.7 [NR]	NR	acute: 0.0 late: 0.0 (RTOG/EORTC)
Comito T et al., 2017 [28]	retrosp	isolated Rec	31 [20]	local Rec	Rapidarc FFF	ITV + 5 mm or GTV + 5-7 mm	to mean PTV dose	45/6 Fr.	65.5	95.0	20.0* 77.0 after	SAA: 58.0 RAA: 40.0 [NRS]	NR	acute: 0.0 late: 0.0 (CTCAE V.4.0)
Koong AJ et al., 2017 [29]	retrosp	previously irradiated local Rec	23 [14]	local Rec, M_0	RSBRT or standard LINAC	ITV + 2-3 mm	NR	25/5 Fr.	31.3	40.0	26.1*	"relative improvement": 57.1 [NR]	Increased pain due to local progression: 1 pt (4.5%)	acute: GI (8.7) late: 0.0 (CTCAE v. 4.0)
Zeng XL et al., 2016 [30]	retrosp	local precurrences ± distant	24 [13]	local Rec, ± distant M_1	RSBRT	GTV + 3 mm	95% PTV encompassed by 73% isodose	45(42-50)/ 5(5-8) Fr.	71.3	108.0	100.0*	reduced pain (from 7.2±2.5 to 2.7±1.3) 1 week after SBRT; slight increase of pain 1 day after SBRT (from 7.2±2.5 to 8.9±1.2) [VAS]	Reduced pain after almost 6 months	acute: GI (4.2) late: 0.0 (CTCAE v. 4.0)
Ryan JF et al., 2018 [31]	retrosp	not surgical candidates	29 [11]	LA, M_0	SBRT with standard LINAC	GTV/ITV + 1-3 mm	NR	28 (25-33) > 5 Fr.	36.9	49.3	76.0* 31.0 after	abdominal pain relief: 73.0 [NCI CTCAE]	NR	acute: GI bleed (3.4), abdominal pain (6.9) late: enterocutaneous fistula (3.8) (NCI CTCAE)

Tian Q et al., 2018 [32]	prosp	not surgical candidates	31 [28]	stage III-IV, M_1	RSBRT	GTV + adjacent vasculature without expansion	to 70 – 80% isodose	40 (40-42)/7-10 Fr.	50.0	64.0	12.9*	"significant" improvement of abdominal pain: 57.0 [Brief Pain Inventory]	NR	acute: haematological (29.0) late: ileus (3.2) (CTCAE V.4.0)
Ji K et al., 2018 [33]	prosp	LA	35 [35]	LA	RSBRT+/- celiac block	GTV + 3 mm	NR	40 (35-45)/5 Fr.	60.0	88.0	71.4	significantly reduced pain ($p < 0.05$) [NRS]	NR	acute: 0.0 late: 0.0 (CTCAE V.5.0)
Koong AJ et al., 2020 [34]	retrosp	metastatic	27 [17]	$T_{2-4}N_{0-1}$ M_1 (1-3 mets)	RSBRT or standard LINAC	ITV + 2-3 mm	95% PTV coverage with prescription isodose	25 (25-40)/1 Fr. & 33 (25-40)/ 5 Fr.	72.9** & 45.7	140** & 63.4	93.0* 26.0 after	CR: 30.0 PR: 60.0 RAA: 46.0 (Stanford Pain Scale)	NR	acute or late: 7.4 (NCI CTCAE)

Abbreviations: 3D-CRT: 3D-conformal radiation therapy; CC: cranio-caudally; CR: complete response; CTV: clinical target volume; EORTC: European Organisation for Research and Treatment of Cancer; GI: gastrointestinal; FFF: filter flattering free; Fr.: fractions; FR.GTV: gross tumor volume; ICRU: international commission on radiation units; ITV: internal tumor volume; LA: locally advanced; M: metastases; N: nodes; NR: not reported; NRS: numerical rating scale; OME: Oral morphine-equivalents; PR: partial response; PTV: planning target volume; RAA: reduction of analgesic administration; Rec: recurrence; retrosp: retrospective; RRS: robotic radiosurgery; RSBRT: robotic stereotactic radiotherapy; RTOG: radiation therapy oncology group; SAA: suspension of analgesic administration; SF: single fraction; VMAT: volumetric modulated arc therapy; WHO: world health organization; *: induction chemotherapy; †: previous RT: 34.1%; ‡: only patients with moderate/severe pain; §: prior RT: 21%; ||: 10 patients received SBRT as a boost after chemoradiation; ¶: elevated aspartate/alanine aminotransferase: 10%; **: 17 received 25 Gy single fraction.

Excluded studies after full text assessment

1. Rao AD, Sugar EA, Chang DT, Goodman KA, Hacker-Prietz A, Rosati LM, Columbo L, O'Reilly E, Fisher GA, Zheng L, Pai JS, Griffith ME, Laheru DA, Iacobuzio-Donahue CA, Wolfgang CL, Koong A, Herman JM. Patient-reported outcomes of a multicenter phase 2 study investigating gemcitabine and stereotactic body radiation therapy in locally advanced pancreatic cancer. Pract Radiat Oncol. 2016;6:417-424. (<u>duplicate data</u>)
2. Perkins CL, El-Reyes B, Simon E, Kooby D, Torres W, Kauh JS, Staley CA, Landry JC. Single-fraction image-guided extracranial radiosurgery for recurrent and metastatic abdominal and pelvic cancers: short-term local control, metabolic response, and toxicity. J Gastrointest Oncol. 2010;1:16-23. (<u>data on pancreatic cancer not reported separately</u>)
3. Scorsetti M, Bignardi M, Alongi F, Fogliata A, Mancosu P, Navarria P, Castiglioni S, Pentimalli S, Tozzi A, Cozzi L. Stereotactic body radiation therapy for abdominal targets using volumetric intensity modulated arc therapy with RapidArc: feasibility and clinical preliminary results. Acta Oncol. 2011;50:528-38. (<u>data on pancreatic cancer not reported separately</u>)
4. Zhu X, Li F, Shi D, Ju X, Cao Y, Shen Y, Cao F, Qing S, Fang F, Jia Z, Zhang H. Health-related quality of life for gemcitabine and nab-paclitaxel plus radiotherapy versus gemcitabine and S-1 plus radiotherapy in patients with metastatic pancreatic cancer. Cancer Manag Res. 2018 Oct 23;10:4805-4815. (<u>patients not completing the prescribed radiotherapy or chemotherapy were excluded; patients with local or distant progression within treatment period were excluded</u>)

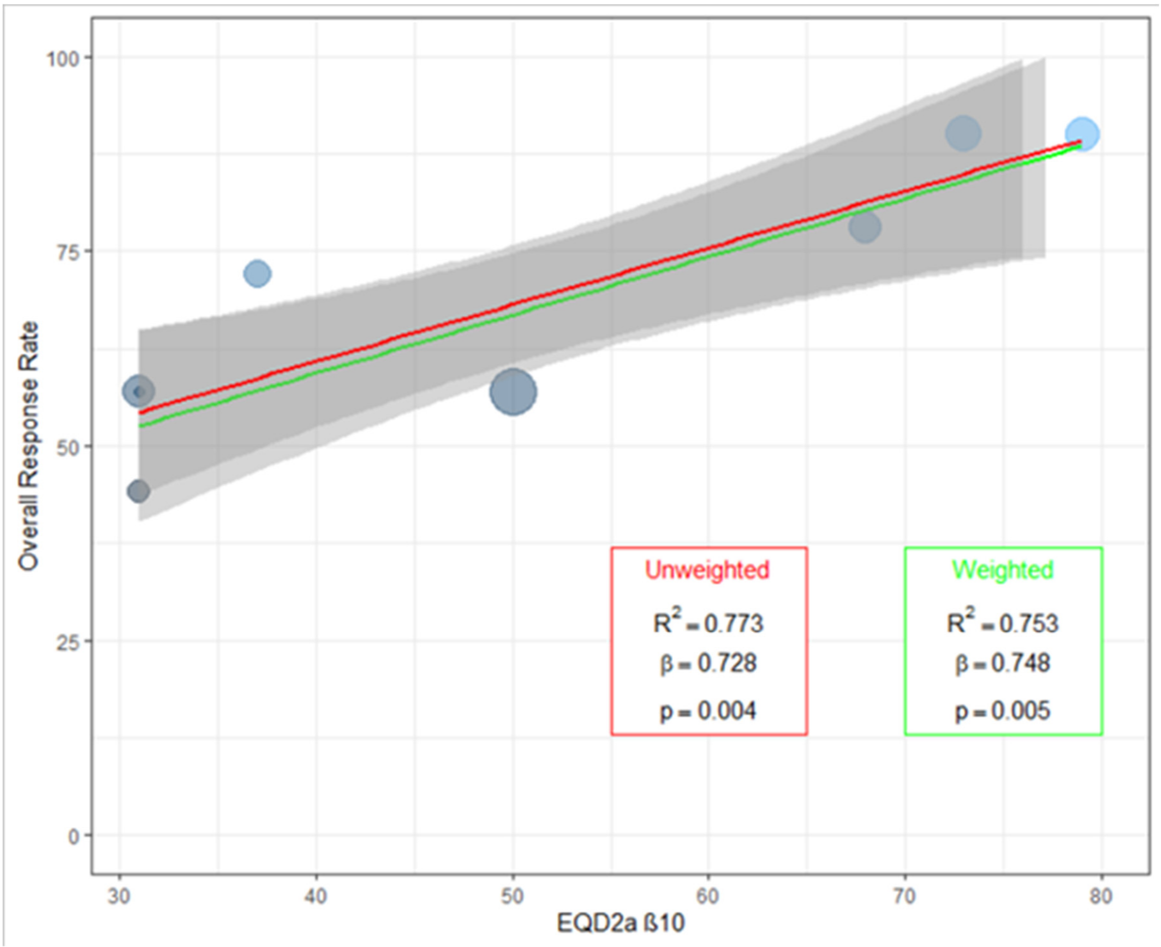

Figure S1. linear regression with sensitivity analysis (removal of an outlier: Didolkar et al. 2010) of equivalent total doses in 2-Gy fractions versus overall response rate of pain.
